# Supplementary material for: Influence of large open-pit mines on the construction and optimization of urban ecological networks: A case study of Fushun City, China
Source: PLoS One. 2024 Jun 27;19(6):e0303016. doi: 10.1371/journal.pone.0303016 (PMC11210778; doi:10.1371/journal.pone.0303016)
Supplement: Attachment — Submitted filename: Response to Reviewers.docx [file pone.0303016.s002.docx]

Response to "Journal Requirements" and "Additional Editor Comments"：

***Question about "Journal Requirements"***
Please review your reference list to ensure that it is complete and correct.
***Question about "Additional Editor Comments"***
Dear authors, please, check for references not found in the revision version R1

***Response to the above questions***

Due to our citation of references in the process of the wrong operation led to the citation reported error, we have made changes to the citation of references, and there is no withdrawn paper.

Along with the modification of the submission, we uploaded the figure files to the Preflight Analysis and Conversion Engine (PACE) digital diagnostic.
